# Supplementary material for: Nature-Based Interventions for Psychological Wellbeing in Long-Term Conditions: A Systematic Review
Source: Int J Environ Res Public Health. 2022 Mar 9;19(6):3214. doi: 10.3390/ijerph19063214 (PMC8954238; doi:10.3390/ijerph19063214)
Supplement: Supplementary file 1 [file ijerph-19-03214-s001.zip › ijerph-1588650-supplementary.pdf]

Supplementary Table S1: Quality assessment ratings using BestBETS assessment checklist for cohort healthcare research.

**Author (year)**	1.1 Are the objectives of the study clearly stated?	2.1 Is the study design suitable for the objectives	2.2 Who/what was studied?	2.3 Was this the right sample to answer the objectives?	2.4 Is the study large enough to achieve its objectives? Have sample size estimates been performed?	2.5 Were all subjects accounted for?	2.6 Were all appropriate outcomes considered?	2.7 Has ethical approval been obtained if appropriate?	2.8 Were the patients randomised between treatments?	2.9 How was randomisation carried out?	2.10 Are the outcomes clinically relevant?	3.1 Is it clear what was measured, how it was measured and what the outcomes were?	3.2 Are the measurements valid?	3.3 Are the measurements reliable?	3.4 Are the measurements reproducible?	3.5 Were the patients and the investigators blinded?	4.1 Are the basic data adequately described?	4.2 Were groups comparable at baseline?	4.3 Are the results presented clearly, objectively and in sufficient detail to enable readers to make their own judgement?	4.4 Are the results internally consistent, i.e. do the numbers add up properly?	4.5 Were side effects reported?	5.1 Are the data suitable for analysis?	5.2 Are the methods appropriate to the data?	5.3 Are any statistics correctly performed and interpreted?	6.1 Are the results discussed in relation to existing knowledge on the subject and study objectives?	6.2 Is the discussion biased?	7.1 Are the authors' conclusions justified by the data?	7.2 What level of evidence has this paper presented? (using CEBM levels)	7.3 Does this paper help me answer my problem?	8.1 Can any necessary change be implemented in practice?	8.2 What aids to implementation exist?	8.3 What barriers to implementation exist?	**TOTAL score**
Beinotti et al. (2013)	2	2	2	2	1	2	2	2	2	0	2	2	2	2	2	2	2	2	2	2	0	2	2	1	2	2	1	2b	2	2	1	1	**53**
Chun et al. (2017)	2	2	2	2	2	2	2	2	2	2	2	2	2	2	2	1	2	2	2	2	0	2	2	2	2	2	2	2b	2	2	1	1	**57**
Jia et al. (2016)	2	2	2	2	1	1	2	2	2	0	2	2	2	2	2	1	1	2	1	0	0	2	2	2	2	2	2	2b	2	2	1	1	**49**
Song et al. (2015)	2	2	2	2	1	2	2	2	2	0	2	2	2	2	2	1	2	0	1	0	0	2	2	1	2	2	1	2b	2	2	1	1	**47**
Li et al. (2016)	2	2	2	2	1	2	2	2	1	N/A	2	2	2	2	2	1	2	N/A	2	2	0	2	2	1	2	2	1	3b	1	2	1	1	**48**
Ochiai et al. (2015)	2	2	2	2	1	2	2	2	1	N/A	2	2	2	2	2	1	2	N/A	1	1	0	2	2	1	2	2	2	3b	2	2	1	1	**48**
Song et al. (2017)	2	2	2	2	1	2	2	2	2	0	2	2	2	2	2	1	1	N/A	1	2	0	2	2	1	2	2	2	2b	2	2	1	1	**49**
Sung et al. (2012)	2	2	2	2	2	2	2	2	1	N/A	2	2	2	2	2	1	2	2	1	2	0	2	2	1	2	2	2	2b	2	2	1	1	**52**
Wu et al. (2020)	2	2	2	2	2	2	2	2	2	0	2	2	2	2	2	1	2	2	1	1	0	2	2	1	2	2	2	2b	2	2	1	1	**52**
Mao et al. (2012)	2	2	2	2	1	2	2	2	2	0	2	2	2	2	2	1	1	2	1	1	0	2	2	1	2	2	2	2b	2	2	1	1	**50**
Mao et al. (2017)	2	2	2	2	2	2	2	2	2	0	2	2	2	2	2	1	1	2	1	1	0	2	2	1	2	2	2	2b	2	2	1	1	**51**
Wichrowski et al. (2005)	2	2	2	2	2	2	2	0	1	N/A	2	2	2	2	2	1	2	2	1	1	0	2	2	1	2	2	2	2b	2	2	1	1	**49**

0 Not reported 1 Reported but inadequate 2 Reported and adequate
